# Expansion of Functional Myeloid-Derived Suppressor Cells in Controlled Human Malaria Infection

**DOI:** 10.3389/fimmu.2021.625712

**Published:** 2021-03-19

**Authors:** Carlos Lamsfus Calle, Rolf Fendel, Anurag Singh, Thomas L. Richie, Stephen L. Hoffman, Peter G. Kremsner, Benjamin Mordmüller

**Affiliations:** ^1^Institute of Tropical Medicine, University of Tübingen, Tübingen, Germany; ^2^German Center for Infection Research (DZIF), Partner Site Tübingen, Tübingen, Germany; ^3^Centre de Recherches Médicales de Lambaréné (CERMEL), Lambaréné, Gabon; ^4^Department of Pediatrics 1, University Children's Hospital Tübingen, Tübingen, Germany; ^5^Institute for Clinical and Experimental Transfusion Medicine, University Hospital Tübingen, Tübingen, Germany; ^6^Sanaria Inc., Rockville, MD, United States

**Keywords:** *Plasmodium falciparum*, immunosuppression, myeloid-derived suppressor cells, PfSPZ-CVac, malaria vaccine, PfSPZ vaccine, controlled human malaria infection, regulatory T cell

## Abstract

Malaria can cause life-threatening complications which are often associated with inflammatory reactions. More subtle, but also contributing to the burden of disease are chronic, often subclinical infections, which result in conditions like anemia and immunologic hyporesponsiveness. Although very frequent, such infections are difficult to study in endemic regions because of interaction with concurrent infections and immune responses. In particular, knowledge about mechanisms of malaria-induced immunosuppression is scarce. We measured circulating immune cells by cytometry in healthy, malaria-naïve, adult volunteers undergoing controlled human malaria infection (CHMI) with a focus on potentially immunosuppressive cells. Infectious *Plasmodium falciparum* (Pf) sporozoites (SPZ) (PfSPZ Challenge) were inoculated during two independent studies to assess malaria vaccine efficacy. Volunteers were followed daily until parasites were detected in the circulation by RT-qPCR. This allowed us to analyze immune responses during pre-patency and at very low parasite densities in malaria-naïve healthy adults. We observed a consistent increase in circulating polymorphonuclear myeloid-derived suppressor cells (PMN-MDSC) in volunteers who developed *P. falciparum* blood stage parasitemia. The increase was independent of preceding vaccination with a pre-erythrocytic malaria vaccine. PMN-MDSC were functional, they suppressed CD4^+^ and CD8^+^ T cell proliferation as shown by *ex-vivo* co-cultivation with stimulated T cells. PMN-MDSC reduced T cell proliferation upon stimulation by about 50%. Interestingly, high circulating PMN-MDSC numbers were associated with lymphocytopenia. The number of circulating regulatory T cells (T_reg_) and monocytic MDSC (M-MDSC) showed no significant parasitemia-dependent variation. These results highlight PMN-MDSC in the peripheral circulation as an early indicator of infection during malaria. They suppress CD4^+^ and CD8^+^ T cell proliferation *in vitro*. Their contribution to immunosuppression *in vivo* in subclinical and uncomplicated malaria will be the subject of further research. Pre-emptive antimalarial pre-treatment of vaccinees to reverse malaria-associated PMN-MDSC immunosuppression could improve vaccine response in exposed individuals.

## Introduction

Malaria is responsible for ~409,000 deaths, resulting from about 229 million new cases of malaria per year globally (1). A steady decrease in malaria-associated morbidity and mortality was achieved due to implementation of malaria control efforts applied since the beginning of this century. However, from 2015 the decrease in the burden of malaria has stalled. The development of an effective malaria vaccine has become a research priority (1–4).

In malaria-endemic areas, a large fraction of infections in older children and adults is asymptomatic or remains unnoticed. Regardless of malaria prevalence being high or low, subclinical parasitemias should not be neglected when aiming for elimination, as they also contribute strongly to ongoing transmission (5–12). Chronic low parasitemias with no or mild signs and symptoms can still have a strong impact, mostly through the development of anemia and suppression of immune responses against vaccines and other infectious diseases (13–16). Both consequences of chronic plasmodial infections often remain unrecognized despite having an important long-lasting impact, particularly in children (17–19). Mechanisms that lead to suppressed immune responses during malaria, convalescence and asymptomatic infections in humans are poorly understood and difficult to investigate in endemic areas. Especially, co-infections, high and variable malaria incidence, as well as other genetic and environmental factors, impede a systematic analysis, and therefore large cohorts are required to circumvent these issues.

In healthy malaria-naïve European volunteers, a smaller cohort is sufficient when a standardized controlled human malaria infection (CHMI) model is used. Therefore, we took advantage of this model of subclinical parasitemia to study the kinetics of potential immunosuppressive cells in naïve individuals. CHMI is done by direct venous inoculation (DVI) of 3.2 × 10^3^ purified, cryopreserved fully infectious *Plasmodium falciparum* (Pf) sporozoites (SPZ) (PfSPZ Challenge) (20) and has become a gold standard in the field for assessing vaccine candidates (21), chemopreventive drugs (22) and naturally acquired immunity (23, 24). Advantages of standardized CHMI are that volunteers are prospectively chosen, start and dose of the infection are defined, infection rate is practically 100% with intense follow-up. The infection is stopped based on parasitemia thresholds and clinical criteria, often below the symptomatic threshold.

Chronic and subclinical as well as low-parasitemic Plasmodium infections do not induce strong inflammatory responses in endemic settings (8, 25). Upon repeat exposure to the parasite, anti-disease immunity develops first, followed by anti-parasitic immunity that can be highly effective (26–28) – up to sterile immunity in a small percentage of individuals (29, 30). In contrast to sterile immunity, anti-disease immunity is characterized by developing tolerance to parasitemia (31), which can be associated with a more general immunosuppression (32). To date, CD4^+^ regulatory T cells (T_reg_) have been the main focus of studies to investigate the mechanism of immunological tolerance by immunosuppression during malaria (33–38). T_reg_ block T cell responses by inhibitory cytokines (membrane-bound or pericellular), cytolysis, cellular metabolic disruption and modulating dendritic cells through co-stimulatory markers (39–42). Interestingly, dendritic cells may also contribute to immunosuppression (43–45).

Myeloid-derived suppressor cells (MDSC) are a heterogeneous population of regulatory immature myeloid cells that suppress immune responses mainly by blocking T cell responses (46–48). MDSC are induced and maintained by inflammatory mediators and can contribute to the support of chronic infections and support T_reg_ development (49). Activated by pathogen molecules, their regulatory activities depend not only on cell to cell contact, but also on the production of some characteristic extracellular factors (48). MDSC can be stratified into two major phenotypes: (i) polymorphonuclear MDSC (PMN-MDSC) and (ii) monocytic MDSC (M-MDSC), whose circulating kinetics reflect and predict the clinical outcome in different diseases (46, 50, 51). Despite their immune-regulatory capacities and their close contact with parasites in the blood, the presence of MDSC during the course of malaria has not been systematically investigated yet (52). Here, we describe for the first time the peripheral blood kinetics of MDSC within the highly controlled setting of a malaria infection model. Resulting alterations in MDSC homeostasis during infection may be a yet unknown mechanism for *P. falciparum*-induced immune tolerance.

## Materials and Methods

### Participants

All samples were obtained from two independent clinical trials in which efficacy of Sanaria® PfSPZ Vaccine [radiation attenuated, aseptic, purified, cryopreserved *P. falciparum* (Pf) sporozoites (SPZ)] and PfSPZ-CVac (infectious, aseptic, purified, cryopreserved PfSPZ administered with an antimalarial drug) were assessed by CHMI (ClinicalTrials.gov identifiers NCT02858817 and NCT02704533). The studies were conducted at the Institute of Tropical Medicine, University Hospital Tübingen and full results of both trials will be reported elsewhere [Mordmüller et al. unpublished data and (53)].

For the present study on immunosuppressive cell populations, only samples during CHMI were used, regardless of previous vaccination. In trial NCT02858817, a PfSPZ-CVac study, volunteers received either PfSPZ or normal saline (placebo) and atovaquone/proguanil (1,000 /400 mg) (A/P) three times every 4 weeks. CHMI of these subjects immunized with PfSPZ-CVac (A/P) was performed 10 weeks following the last vaccination. Immunizations consisted of three doses of either 5.12 × 10^4^ or 15 × 10^4^ PfSPZ. The second trial (NCT02704533) used PfSPZ Vaccine, which has been described before (54), for immunization. Here, volunteers were vaccinated with either three doses of 9 × 10^5^ or two doses of 1.35 × 10^6^ or 2.7 × 10^6^ PfSPZ of PfSPZ Vaccine. CHMI was done 3 weeks following immunization.

**Figure 1:**
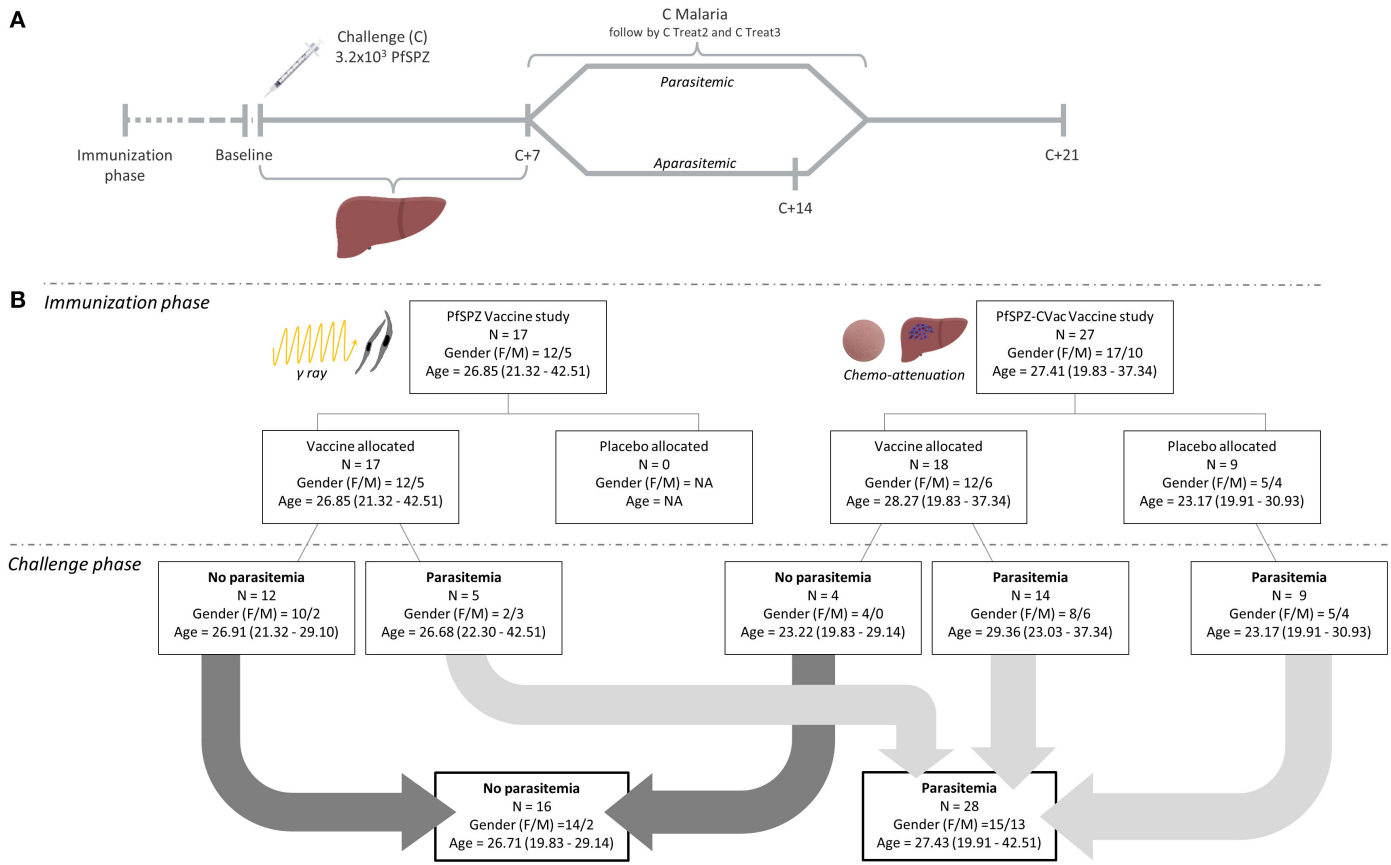
Study timeline and study outcome overview. **(A)** The timeline represents the overview of the blood samples to investigate the kinetics of immunosuppressive cells during the controlled human malaria infection (CHMI) in the challenge phase of the malaria vaccine trials. Baseline: before the DVI of 3.2 × 10^3^ fully infectious PfSPZ Challenge (C); C+7: 7 days after injection; C Malaria: parasitemic after fulfilling the treatment initiation end-point criteria; C Treat2 and C Treat3: subsequent days 2 and 3 of malaria treatment. C+14: 14 days after DVI for study volunteers not developing any parasitemia during CHMI; C+21: at the end of CHMI, 21 days after DVI. **(B)** The chart outlines detailed information on the number of participants vaccinated with either PfSPZ Vaccine or PfSPZ-CVac vaccine during the immunization phase and the respective outcome of the vaccine trials during the challenge phase. All groups are stratified by sex, age, vaccine and study arm. Age is reported in years as median and range; and sex as female/male (F/M) ratio.

CHMI was performed with 3.2 × 10^3^ sporozoites [Sanaria® PfSPZ Challenge (PfNF54)] by DVI. Volunteers had daily clinical visits, thick blood smear readings and reverse transcription quantitative PCR (RT-qPCR) for up to 3 weeks. RT-qPCR was performed as described previously, achieving a lower limit of detection of six parasites/ml (55). The end-point for treatment initiation with atovaquone/proguanil was met when a volunteer became parasitemic, defined as three consecutive positive RT-qPCR results with at least one parasitemia above 100 parasites per ml or any parasitemia detected by microscopy. Peripheral blood mononuclear cells (PBMC) were prepared in the following sampling days during CHMI (Figure 1A). (i) Baseline, before the injection of fully infectious PfSPZ Challenge (C); (ii) C+7, generally the day of first detectable parasitemia by RT-qPCR; (iii) C Malaria, when parasitemic participants fulfilled the treatment initiation end-point criteria, being followed by two more samples at the 2nd (C Treat2) and 3rd (C Treat3) day of treatment; (iv) C+14, in protected aparasitemic participants as comparable control for malaria positive cases; (v) C+21, at the end of CHMI. As per-protocol, participants received a 3 days course treatment with atovaquone/proguanil, administered always after blood sampling at C Malaria, C Treat2 and C Treat3; and C+21 if they were not positive for parasitemia during CHMI.

### MDSC and Regulatory T Cells Characterization

In brief, whole blood was collected in Na-heparin tubes (S-Monovette, Sarstedt) from all volunteers. All samples for analysis were acquired before participants received the first dose of rescue treatment except for those on 2nd and 3rd day of malaria-specific treatment (Figure 1A). As previously described, MDSC and T_reg_ were promptly quantified (56–58) from peripheral blood mononuclear cells (PBMC) freshly isolated by density gradient centrifugation (Ficoll-Paque^TM^ PLUS; GE Healthcare).

MDSC were characterized by flow cytometry (FACScalibur Flow cytometer, BD) after staining PBMC with: mouse anti-human CD14-FITC (BD Biosciences, Clone MϕP9), mouse anti-human HLA-DR-PerCP (BD Biosciences, Clone L243), mouse anti-human CD33-PE (Miltenyi Biotec, Clone AC104.3E3) and rat anti-human CD11b-APC (Miltenyi Biotec) to identify Monocytic-MDSC (M-MDSC) from the monocyte population as CD11b^+^CD33^+^HLA-DR^−/*lo*^CD14^+^; while PBMC in the SSC^hi^ area were defined as PMN-MDSC by the staining with mouse anti-human CD66b-FITC (BD Pharmingen). The percentage of PMN-MDSC was verified from the SSC^hi^ region of the M-MDSC sample by gating the CD11b^+^CD33^+^HLA-DR^−/*lo*^CD14^−^ population thereof (Supplementary Figures 1, 2A,B).

The MDSC counts were normalized to the initial values within participant, defined as the MDSC fold change for every measured time point in relation to the baseline MDSC levels for each individual participant.

Regulatory T cells were defined as surface CD4^+^, CD25^+/high^ and intracellular FoxP3^+^ (Supplementary Figure 2C). Antibody clones used for the staining were mouse anti-human CD4-FITC (BD, Clone SK3), mouse anti-human CD25-PE (BD Pharmingen, Clone M-A251) and anti-human FoxP3-Alexa Fluor 647 (Biolegend, Clone MOPC-21). To control for unspecific staining, the respective isotype control was used (Biolegend). Intracellular staining was performed using a commercial kit following the respective protocol (Cytofix/Cytoperm, BD).

### MDSC Isolation and T Cell Suppression Assay

Peripheral blood mononuclear cells (PBMC) from a healthy donor were stained with arboxyfluorescein diacetate succinimidyl ester [Vybrant® CFDA SE (CFSE) Cell Tracer Kit, #V12883, Molecular probes by life technologies] according to the manufacturer's manual allowing lymphocyte proliferation traceability by their simultaneous stimulation with 100 U/ml IL-2 (R&D Systems) and 1 μg/ml purified NA/LE Mouse anti-human CD3 (BD Pharmingen, #555336, Clone HIT3a).

PMN-MDSC were freshly isolated from parasitemic participant's PBMC (autoMACS®Pro Separator, Miltenyi Biotec), gathered by positive selection after mouse anti-human CD66b-FITC (BD Pharmingen, #555724) and anti-FITC MicroBeads (Miltenyi Biotec, #130-048-701) staining, as described before (59). Subsequently, the highly purified PMN-MDSC were seeded in a round bottom 96-well plate in different proportions to a fixed amount of 6 × 10^4^ CFSE labeled PBMC per well (1:2, 1:4, 1:8, 1:16) and incubated at 37°C and 5% CO_2_. Complete media (RPMI1640 supplemented with 10% autologous serum, 1% L-glutamine, and 1% Penicillin-Streptomycin) without additional cells was added to stimulated and non-stimulated labeled PBMC as positive and negative control, respectively. As controls for the contribution of polymorphonuclear cells (PMN) to suppression, PMN from the participants isolated with an erythrocyte lysis step from the high-density fraction of the Ficoll were seeded in the same manner as PMN-MDSC.

After 4 days of incubation, cells were harvested and stained with mouse IgG1, κ anti-human CD4-PE (Biolegend, #300508, Clone RPA-T4) and mouse IgG1, κ anti-human CD8a-APC (Biolegend, #300912, Clone HIT8a). Shortly before measurement, Propidium Iodide (BD Pharmingen, #51-66211E) was added to determine cell viability. CFSE fluorescence intensity was analyzed by flow cytometry to determine proliferation of CD4^+^ and CD8^+^ T cells (Supplementary Figures 2D,E). Proliferation was defined as the ratio of percentage of T cell proliferated following addition of PMN-MDSC or PMN to percentage of T cell proliferation without co-culture (58).

### Determination of Lymphocytopenia

The hematological parameters from participants' blood samples were measured with the Sysmex XN-series hematology analyzer. Lymphocytopenia was defined according to the central laboratory reference range (lymphocyte cell count ≤ 1.2 × 10^3^/μl of blood).

### Statistical Analysis

Flow Cytometry data was analyzed using FlowJo v10.6.1. Statistical tests were performed using GraphPad Prism 8 (GraphPad Software, La Jolla, CA, USA). Figures were created using ggplot2 package version 3.3.0 in R software (R-project, https://www.R-project.org/) version 3.6.3.

The area under the curve was computed by using the trapezoid rule.

Multiple comparisons on cellular kinetics were calculated by mixed effects (Model III) ANOVA where factors are both fixed and random. This approach represents a normal ANOVA when some random missing values are presented. As the experiment design is repeated measures, sphericity was not assumed, following the recommendation of Maxwell and Delaney. The mixed model uses a compound symmetry covariance matrix, and is fit using Restricted Maximum Likelihood (REML). Variation analysis was corrected for multiple comparisons by the “two-stage” Benjamini et al. procedure for controlling the false discovery rate (FDR) (60).

## Results

### CHMI Outcome From Vaccine Studies

Circulating MDSC kinetics were quantified during CHMI, done in a subset of participants of the two clinical malaria vaccine trials (trial identifiers: NCT02704533, NCT02858817) in healthy naïve volunteers that will be reported elsewhere [Mordmüller et al. unpublished data and (53)]. A total of 44 participants underwent CHMI as described (20). Ten to 3 weeks before CHMI, volunteers completed immunizations with either placebo (normal saline; *n* = 9), PfSPZ Vaccine (*n* = 17) or PfSPZ-CVac (A/P) (*n* = 18) (Figure 1B). PfSPZ Vaccine and PfSPZ-CVac (A/P) lead to a complete arrest of parasite development during liver stage. As expected, none of the participants developed blood stage parasitemia detectable by ultra-sensitive RT-qPCR during the immunization phase.

A total of 28 out of the 44 (64 %) participants undergoing CHMI by DVI administration of PfSPZ Challenge developed parasitemia and received a 3-day course treatment with atovaquone/proguanil. Of those 28 unprotected volunteers, five were part of the PfSPZ Vaccine study and 23, including the nine placebos, were part of PfSPZ-CVac (A/P) study. The geometric mean of days from DVI to rescue treatment in participants with circulating parasites was 11.7 days (range 10–14 days). None of the protected participants had parasites detectable in the circulation.

The highest detectable parasitemia in parasitemic participants during CHMI was 12 parasites per μl. The most frequent adverse event (AE) reported at this stage was headache (Supplementary Table 1). Notably one participant did not report any AE when study treatment end-point was reached. Elevated temperature of 38°C was observed in only one infected participant and occurred on the second treatment day. Furthermore, during the CHMI phase the severity of the AE was not dependent on infection on the days the AE were detected (Chi-square independence test, *p* = 0.8823). However, the number of reported AE increased substantially during the 2nd and 3rd day of treatment. Among the most common AE, lymphocytopenia stood out in parasitemic participants during the treatment days, which is described in more detail in a separate section below. In summary, the CHMI led to a very early state of malaria infection with no or few mild symptoms and signs.

### Suppressor Cells Kinetics in CHMI

**Figure 2:**
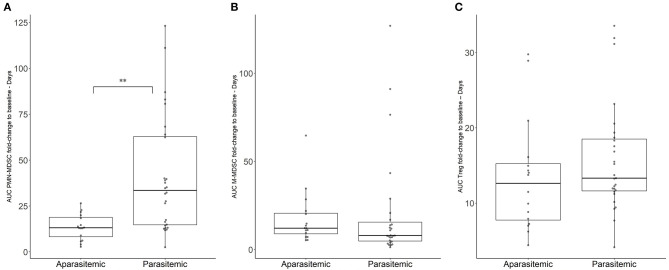
Variation of immunosuppressive cell populations in blood during CHMI. Immunosuppressive cell populations were estimated by flow cytometry. Area under the cell FC to baseline-days curve (AUC) was estimated for **(A)** PMN-MDSC, **(B)** M-MDSC, **(C)** T_reg_. Boxplots reflect median, interquartile range as well as the min/max range. The significant difference between AUC in parasitemic and aparasitemic was specifically labeled (**p < 0.001).

Circulating suppressor cells were phenotypically characterized in participants' blood during CHMI. Cellular variation of circulating suppressor cells in the PBMC of the participants was controlled by adjusting all levels to the baseline levels within the same subjects prior to injection of the PfSPZ Challenge (Figure 2). The resulting area under the PMN-MDSC fold change increase (FC) to baseline - day curve (AUC) for each participant reflects the kinetic expansion of PMN-MDSC over the CHMI days, given in the unit of FC-days. Therefore, the median for the AUC was 35 [interquartile range (IQR), 15–64] FC-days in parasitemic participants, which was significantly higher than the 13 (IQR, 8–18) FC-days from the aparasitemic group (Figure 2A, *p* < 0.0001). This represents a general increase in circulating PMN-MDSC in those participants developing blood stage parasitemia. PMN-MDSC kinetics are similar between the different doses of parasites received during the previous immunization phase (*p* = 0.07; Supplementary Figure 3A), nor by being allocated to placebo or vaccine (*p* = 0.37; Supplementary Figure 3B), but by the development of detectable parasites in the circulation.

The kinetics of the other measured suppressor cells did not change to the same extent. The median AUC of 12 (IQR, 7–20) FC-days for the M-MDSC kinetics in the aparasitemic group was not significantly different from the 8 (IQR, 5–17) FC-days of the parasitemic group (Figure 2B, *p* = 0.4552). In the same fashion, the regulatory T cells (T_reg_) median AUC was 13 (IQR, 12–19) T_reg_ FC-days for the parasitemic group and 14 (IQR, 8–16) FC-days for the aparasitemic individuals (Figure 2C, *p* = 0.36). Although the FC increase in Treg was not found to be significantly different between parasitemic and aparasitemic participants, a significant increase of Treg in C Malaria could be detected when the increase of Treg in the non-protected individuals from baseline to day of treatment was analyzed (One-way ANOVA, *p*-value < 0.001; mixed-effect model Benjamini, Krieger and Yekutieli *post-hoc* test Baseline vs. C Malaria *p* = 0.006, Supplementary Figure 4).

**Figure 3:**
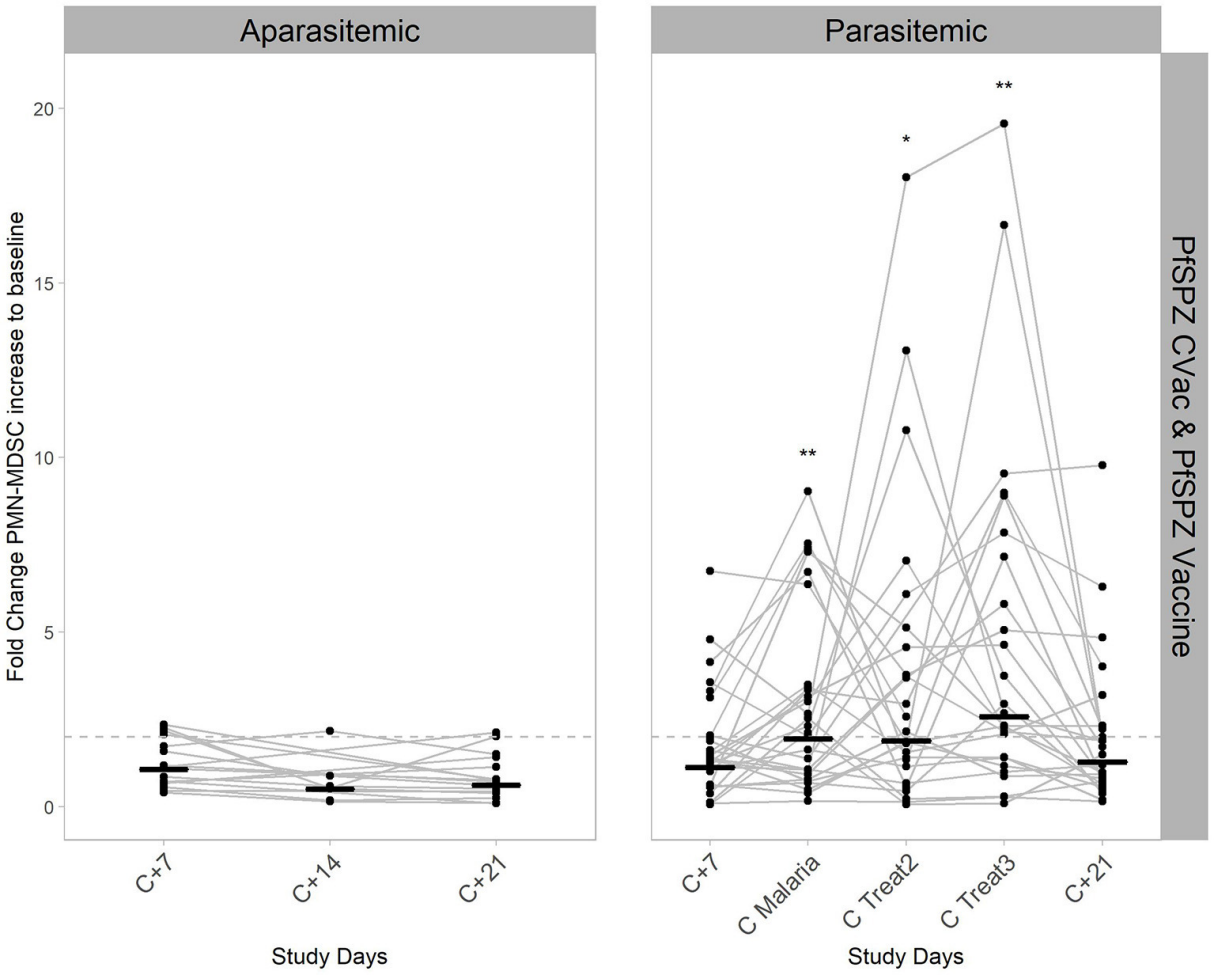
Kinetics of PMN-MDSC during CHMI. PMN-MDSC were estimated in the peripheral blood by flow cytometry, both in aparasitemic (**left**) and parasitemic (**right**) individuals. Data shown, includes the participants from both vaccine studies, PfSPZ-CVac (A/P) and PfSPZ Vaccine. Lines connecting dots show the PMN-MDSC fold change from baseline for every individual from days C+7 to C+21 after injection of viable PfSPZ Challenge. Short black horizontal lines represent the geometric mean values of PMN-MDSC fold change from baseline for each time point. The significant variation per day compared to baseline in the parasitemic group is reflected by the *p*-value * < 0.05; ** < 0.005. Dashed line represents 2-fold change relative to baseline.

To more specifically determine the exact time point at which the PMN-MDSC are significantly higher in the parasitemic individuals, a mixed effect approach (Model III - following a two-way ANOVA approach when some random values are missing) in combination with the respective *post-hoc* test was done (Figure 3). The fold changes increase of PMN-MDSC to baseline were tested by using the study days and protection status (defined by the growth of parasites in the blood) as factors for the multiple comparisons. PMN-MDSC kinetics were explained by being unprotected (*p* < 0.001) and by their respective timing (study days) of parasitemia (*p* < 0.001), but the overall change on PMN-MDSC kinetics was not explained by the study days as the standalone factor (*p* = 0.8). The respective *post-hoc* test (Two-stage linear step-up procedure of Benjamini, Krieger and Yekutieli) revealed that circulating PMN-MDSC levels were 4 to 5-fold higher at C Malaria and the two subsequent treatment days in the parasitemic group compared to the aparasitemic counterpart at the respective sampling day C+14 (*q* < 0.001, Figure 3, Table 1). Over time, PMN-MDSC levels increased 1.9–2.5 times compared to baseline in the unprotected individuals (*p* < 0.001, Figure 3, right panel) compared to no change within the group of protected individuals who developed no parasitemia over time (Figure 3, left panel).

**Table 1:** Percentage of PMN-MDSC during CHMI.

**Time point**		**Parasitemic**	**Aparasitemic**	
		**% PMN-MDSC of PBMCs (CI: 95%)**		***p*-value**
Baseline		1.67 (1.10; 2.54)	1.46 (0.79; 2.70)	0.2338
C+7		1.87 (1.25; 2.80)	1.56 (0.95; 2.58)	0.2338
C Malaria*	C+14**	3.25 (2.14; 4.95)*	0.66 (0.42; 1.04)**	**0.0002**
C Treat2*		3.22 (1.99; 5.20)*		**0.0002**
C Treat3*		4.45 (2.91; 6.82)*		** <0.0001**
C+21		2.21 (1.51; 3.22)	0.90 (0.55; 1.47)	**0.0031**

Values are given as the geometric mean of the % PMN-MDSC in PBMCs and their respective 95% confidence interval (CI). P-values are calculated as result of multiple comparison of participants PMN-MDSC % in PBMCs between aparasitemic and parasitemic participants corrected for the different days analyzed. Statistically significant p-values are given as bold values. PMN-MDSC percentage at time points labeled with (*) in parasitemic were compared to the respective control measurement performed at time point C+14 from aparasitemic volunteers (**).

PMN-MDSC levels in the peripheral blood increased significantly and remained elevated for at least the treatment period, which coincides with the appearance of elevated parasite levels in the blood (Supplementary Figure 5). The geometric mean parasitemia at C Malaria, C Treat2 and C Treat3 were 649 parasites/ml (range 4–12,421), 852 parasites/ml (97–11,990) and 68 parasites/ml (3–667), respectively. At C+21, the geometric mean PMN-MDSC level in participants who developed parasites before in CHMI, was still 2.5 times higher (Wilcoxon rank sum test, *p* < 0.005) compared to the PMN-MDSC values on the same day from those who remained aparasitemic (Supplementary Figure 6A). However, at that time, all participants were considered healthy and parasite free, either because they cleared a patent parasitemia after administration of the treatment or because they never developed circulating parasites. As the percentage of PMN-MDSC in the peripheral blood at C+21 correlates with the respective values at C Malaria (Spearman's rank correlation test, *p* = 0.01, *R* = 0.5), the increase might be explained by the strong increase of PMN-MDSC achieved at C Malaria (Supplementary Figure 6B).

### Upregulated PMN-MDSC Suppress CD4^+^ and CD8^+^ T Cell Proliferation *ex vivo*

**Figure 4:**
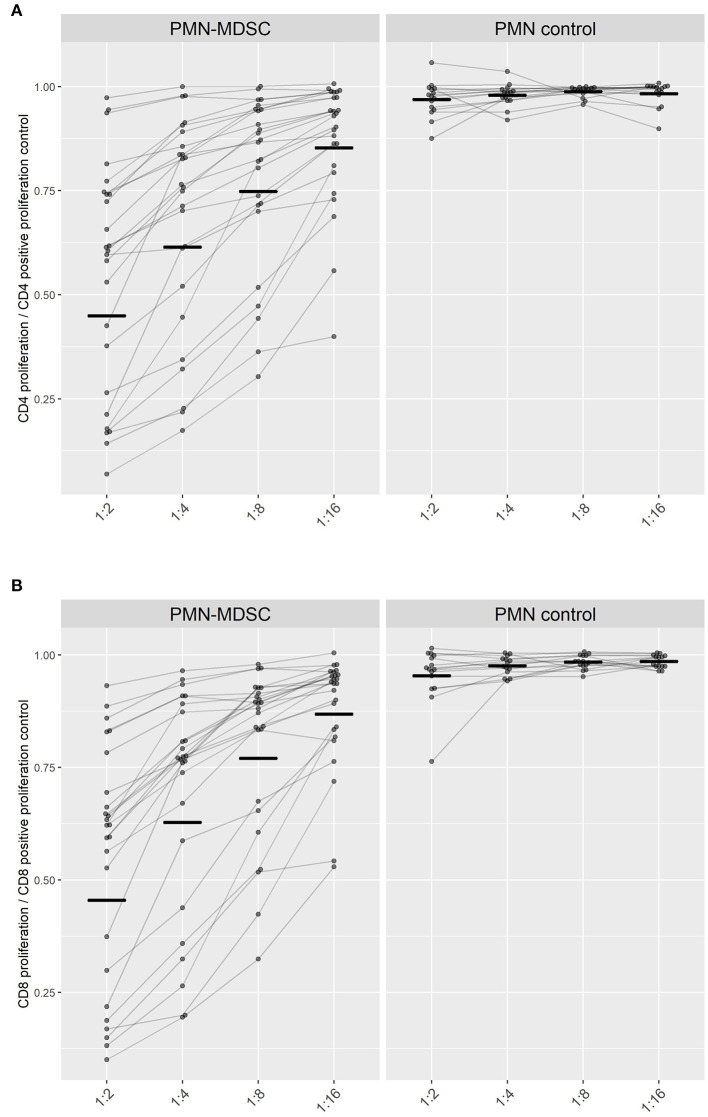
Suppression of T cell proliferation by PMN-MDSC isolated on day C Malaria. The proliferation capacity of **(A)** CD4^+^ T cells and **(B)** CD8^+^ T cells is represented in relation to the maximum proliferation capacity of T cells when incubated alone (set to 1). Ratio of PMN or PMN-MDSC to PBMC were adjusted to 1:2, 1:4, 1:8, 1:16 cells. Paired measurements of the inhibitory capacities of PMN-MDSC (left) or PMN (right) within the same volunteer at different cellular ratios are connected by lines. Black short lines represent the geometric mean for every group.

An *ex-vivo* suppression assay was performed when the end-point criteria for treatment was met at C Malaria in parasitemic participants: 20 out of the 23 participants from the PfSPZ-CVac and all five from the PfSPZ Vaccine study (Figure 4). Thus, the described PMN-MDSC can be distinguished from other non-functional MDSC-like cells expressing similar phenotypic markers. PMN were used in a subgroup of 15 of the 25 participants as controls for the suppression.

PMN-MDSC from parasitemic participants conferred a similar suppression on both CD4^+^- and CD8^+^- T cell-proliferation within the same ratio of PBMC to MDSC seeded (*p*-value > 0.05). The capability of PMN-MDSC to suppress stimulated CD4^+^ and CD8^+^ lymphocytes increased progressively with growing ratios as a linear trend (CD4^+^ & CD8^+^ *p* < 0.0001), reaching at a 1:2 cellular PMN-MDSC to T cell ratio a mean of 54% suppression of lymphoproliferation for both CD4^+^ and CD8^+^ T cells (Figure 4).

Altogether, these findings show that PMN-MDSC found in the circulation during *P. falciparum* parasitemia can suppress CD4^+^ and CD8^+^ T cells proliferation.

### Lymphocytopenia Related to PMN-MDSC Kinetics

**Figure 5:**
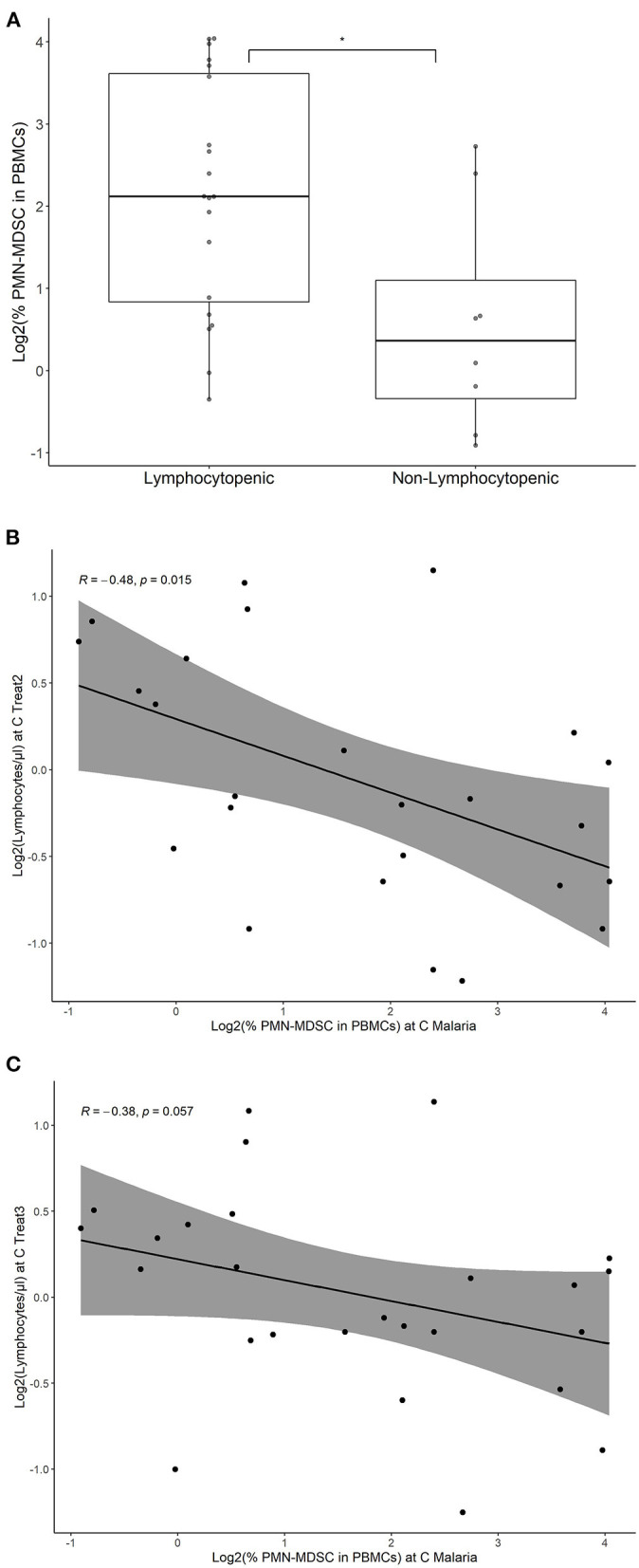
PMN-MDSC at C Malaria in subsequent lymphocytopenia in volunteers with parasitemia. Percentage of PMN-MDSC at C Malaria were estimated by flow cytometry, both in participants developing lymphocytopenia and volunteers not developing lymphocytopenia. Only individuals developing parasitemia during CHMI are shown. **(A)** The individual points represent the fraction of PMN-MDSC in the PBMC for each individual. Boxplots represent the median % PMN-MDSC, the IQR and the lines show the range (min/max) for the two groups. Lymphocytopenia was defined as ≤ 1.2 × 10^3^ lymphocytes per μl. *p < 0.05. Correlation of PMN-MDSC levels at C Malaria and lymphocyte levels at C Treat2 **(B)** and C Treat3 **(C)** are shown. The line represents the linear regression and the gray area represents the 95% confidence interval. Spearman's rank correlation test was performed and the respective correlation coefficients (*R*) and *p*-values were given.

Lymphocytopenia was a common observation in parasitemic participants during the CHMI of both studies. At C Malaria before treatment initiation, the number of circulating lymphocytes in those who are about to develop lymphocytopenia was significantly lower compared to the ones not becoming lymphocytopenic (Figure 5A, Student's *t*-test, *p* < 0.05). However, on treatment initiation day, only four individuals were lymphocytopenic by definition ( ≤ 1.2 × 10^3^/μl). The majority of the parasitemic cases became lymphocytopenic over the second and third treatment days (16 out of 23 in PfSPZ CVac and four out of five in PfSPZ Vaccine).

Logistic regression was used to assess the odds ratio (OR) of becoming lymphocytopenic after treatment initiation with the number of PMN-MDSC at C Malaria. The analysis revealed that increased levels of PMN-MDSC at C Malaria was associated with an increased odds ratio to become lymphocytopenic (OR: 2.3; 95% confidence interval (CI): 1.2–5.5, *p* < 0.05). The PMN-MDSC levels correlated negatively at C Malaria with the subsequent lymphocyte circulating values at C Treat2 and reached borderline significance at C Treat3 (Spearman's rank correlation *p* < 0.05 and *p* = 0.056, respectively, Figures 5B,C), but did not correlate with the lymphocyte levels at C Malaria (*p* = 0.09, data not shown).

Change of lymphocyte counts could be a confounding factor for the presented changes of PMN-MDSC in the peripheral blood. PMN-MDSC are usually represented as the fraction of total PBMC (61). However, we additionally analyzed whether the absolute counts of PMN-MDSC were increased during the treatment period. The analysis revealed that the absolute counts of PMN-MDSC were significantly increased on C Malaria (median increase: 2.1-fold, *p* < 0.01, two-stage linear step-up procedure of Benjamini, Krieger & Yekutieli). After the onset of lymphocytopenia on C Treat2 and C Treat3, numbers of PMN-MDSC were still elevated, but the statistical test did not reach level of significance (median increase: 1.6-fold on both days, *p* > 0.05).

## Discussion

To our knowledge this is the first report of a significant increase in circulating PMN-MDSC during the development of early blood stage parasitemia in human malaria. The expansion of PMN-MDSC was associated with the presence of blood stage parasites in the circulation, even at very low parasitemia; cases of uncomplicated malaria typically have at least 100 times higher parasite density and are detected at much later time points after infection. Interestingly, PMN-MDSC remained elevated days after treatment was completed and no more parasites were detected, which suggests a protracted effect on the immune response. Additional to their phenotypic appearance, PMN-MDSC were confirmed suppressive in an *ex-vivo* assay. Stimulated CD4^+^ and CD8^+^ T cell proliferation was suppressed by the PMN-MDSC collected at a very early stage of malarial infection before any treatment administration.

MDSC drive adaptive and innate immune responses as a homeostatic mechanism to dampen inflammatory responses. Despite their natural physiological role in reestablishing normal steady state after inflammation, when MDSC are elevated in the early stages of pathogen recognition, an inefficient immune response might be generated instead (62). This was also exhibited during CHMI. PMN-MDSC were elevated quite early at the onset of parasitemia, and this could hamper a protective immune response.

PMN-MDSC levels seemed to be independent of earlier exposure to pre-erythrocytic parasite stages during the immunizations before CHMI. The subjects were immunized with malaria vaccines, PfSPZ Vaccine and PfSPZ-CVac (A/P), that do not replicate in hepatocytes (53). Moreover, the pre-erythrocytic parasite stage during CHMI did not lead to an increase in PMN-MDSC count directly after the liver phase on day C+7. Therefore, the main recognized carry-over effect from the immunization phase influencing the PMN-MDSC kinetics is the acquisition of sterile immunity.

Determining the molecular causes of the PMN-MDSC increase during CHMI was not possible with the available data. We were not able to discern whether this was due to direct interaction between the Pf parasites and the PMN-MDSC or due to an indirect physiological reaction initiated by the parasites. Nevertheless, one of the known earliest immune alterations in the CHMI commonly detected after parasites emerge from the liver is the release of TGF-ß in the circulation (63, 64). This cytokine can be activated from its latent form by the blood stage of the parasite, which suggests Pf may be directly involved in an active process in inducing suppressor cell populations (35, 49, 65).

The involvement of anti-inflammatory cytokines early in infection has been previously related to the generation of T_reg_. The kinetics of these cells during infection has been described in mice and human malaria models where T_reg_ are consistently increasing in the circulation after several parasite cycles (35, 63, 66). Because we diagnose and treat in CHMIs based on RT-qPCR positivity, in general the Pf infection concentrations are so low that they are not yet detectable by thick blood smear and not associated with symptoms or only with minor symptoms. In this setting we could not identify a higher proportion of FoxP3+ T_reg_ cells in individuals developing parasitemia in comparison to those not developing parasitemia. Nevertheless, pairwise analysis of the T_reg_ counts at day of treatment to the respective baseline within parasitemic individuals revealed a specific increase of this regulatory cell population. Perhaps a higher and prolonged increase was even missed in our trials, as circulating T_reg_ were not quantified during the second and third day of treatment and malaria treatment was initiated at an early time point before first symptoms arise. Therefore, additional data will be required to determine whether an increase in T_reg_ is following the increase in PMN-MDSC.

Another striking finding is the association of lymphocytopenia, which is a common observation during malaria, with the increase in PMN-MDSC levels. Interestingly, the development of blood stage malaria is known to suppress immune memory responses, reflected in part by an unstable T cell memory (67, 68). Lymphocytopenia has been associated before to a reallocation in the body compartments, however, it might be interesting to check the relevance in immune memory generation in relation to PMN-MDSC (69). In the absence of concomitant natural exposure, sterilizing immunity can be achieved at the liver stage by immunization with attenuated PfSPZ (24, 54, 70–74). However, when the same vaccines and regimens are assessed in populations living in highly endemic areas, the protective efficacy to the vaccines are dramatically reduced (24, 75, 76). Many factors might explain these effects, such as antigenic sin, preexisting antagonistic antibodies and immune dysregulation due to chronic exposure to blood stage parasitemia (77–79), but potentially even more important is suppression of immune responses during immunization by the presence of erythrocytic stage parasitemia (80–82).

The effect of chronic exposure to the parasite on immunosuppressive cells development and maintenance is mainly known for T_reg_. In other diseases, pathogen persistence has been related to the expansion of immunosuppressive PMN-MDSC during infections (50). Some microorganisms succeed in manipulating the suppressive activities of MDSCs by decreasing pathogen recognition capacity and promoting disease chronicity. Remarkably, the PMN-MDSC increased during our studies' CHMI and persisted in the circulation for at least the three treatment-days and likely beyond those days. PMN-MDSC not only tended to be still higher in the unprotected participants at C+21 but also correlated positively with the levels increased at the time when circulating parasites met the criteria for treatment initiation (C Malaria). This finding may be of notice for future vaccine strategies used under recent or constant parasite exposure. For instance, a feedback loop generating an additive effect on MDSC kinetics upon a second infection has been previously described in *Mycobacterium tuberculosis* and HIV infections (83). Repetitive Pf infections in malaria endemic areas, where these co-infections coexist, might have a relevant effect in contributing to an aggravated immune suppression state by the additional generation of MDSC (52).

Unraveling to which extent MDSC kinetics drives the well-known malaria-related suppression may open a new line of investigation. Anyhow, a parasite wash-out period by chemotherapy prior to vaccination might help to generate improved and long-lasting immune responses.

In summary, the early increase of suppressive PMN-MDSC during Pf blood stage infection indicates they are involved in immune regulatory networks. PMN-MDSC may interfere with the generation of immune memory against the parasite as well as with the immune responses against bacterial and viral co-infections. In order to eradicate malaria, not only an effective vaccine but a solid immune system for long-lasting immune memory is needed (84). Even though there is ample evidence of adaptation of parasite survival to human immune responses through immunosuppression, its mechanisms are not yet deciphered, and they seem to be multifactorial. Our data suggest that PMN-MDSC contribute to this regulation.

## Data Availability Statement

The raw data supporting the conclusions of this article will be made available by the authors, without undue reservation.

## Ethics Statement

The trials including the immunological investigations were approved by the ethics committee of the University Hospital in Tübingen. Written informed consent was obtained from all participants in the study. Studies were performed in accordance with the German Medicinal Products Act, the Declaration of Helsinki and ICH-GCP guidelines.

## Author Contributions

CL, BM, and AS conceived the study. CL and RF analyzed the data. CL and AS developed the methodology and performed the experiments. CL drafted the original manuscript. RF and BM contributed to analysis and corrected paper. SH and TR contributed to PfSPZ production, reviewing, and editing of the manuscript. AS, RF, BM, and PK contributed to supervision, reviewing, and editing of the manuscript. All authors contributed to the article and approved the submitted version.

## Conflict of Interest

TR and SH were employed by Sanaria Inc. The remaining authors declare that the research was conducted in the absence of any commercial or financial relationships that could be construed as a potential conflict of interest.

## Abbreviations

CHMI: Controlled human malaria infection
Pf: *Plasmodium falciparum*
SPZ: Sporozoites
PfSPZ: *Plasmodium falciparum Sporozoites*
PfSPZ Challenge, Infectious, aseptic, purified: cryopreserved PfSPZ
PfSPZ Vaccine: Radiation attenuated PfSPZ
PfSPZ-CVac: PfSPZ administered with an antimalarial drug
A/P: Atovaquone/proguanil (1,000 mg/400 mg)
DVI: Direct venous inoculation
T_reg_: CD4^+^ regulatory T cells
MDSC: Myeloid-derived suppressor cells
PMN: Polymorphonuclear cells
PMN-MDSC: Polymorphonuclear myeloid-derived suppressor cells
M-MDSC: Monocytic myeloid-derived suppressor cells
PBMC: Peripheral blood mononuclear cells
CFSE: Carboxyfluorescein diacetate succinimidyl ester
RT-qPCR: Reverse transcription quantitative PCR
AE: Adverse events
FC: Fold change to baseline
AUC: Area under the cell FC-time curve.
