# Supplementary material for: Expansion of Functional Myeloid-Derived Suppressor Cells in Controlled Human Malaria Infection
Source: Front Immunol. 2021 Mar 19;12:625712. doi: 10.3389/fimmu.2021.625712 (PMC8017236; doi:10.3389/fimmu.2021.625712)
Supplement: Supplementary file 1 [file Data_Sheet_1.docx]

Supplementary Material


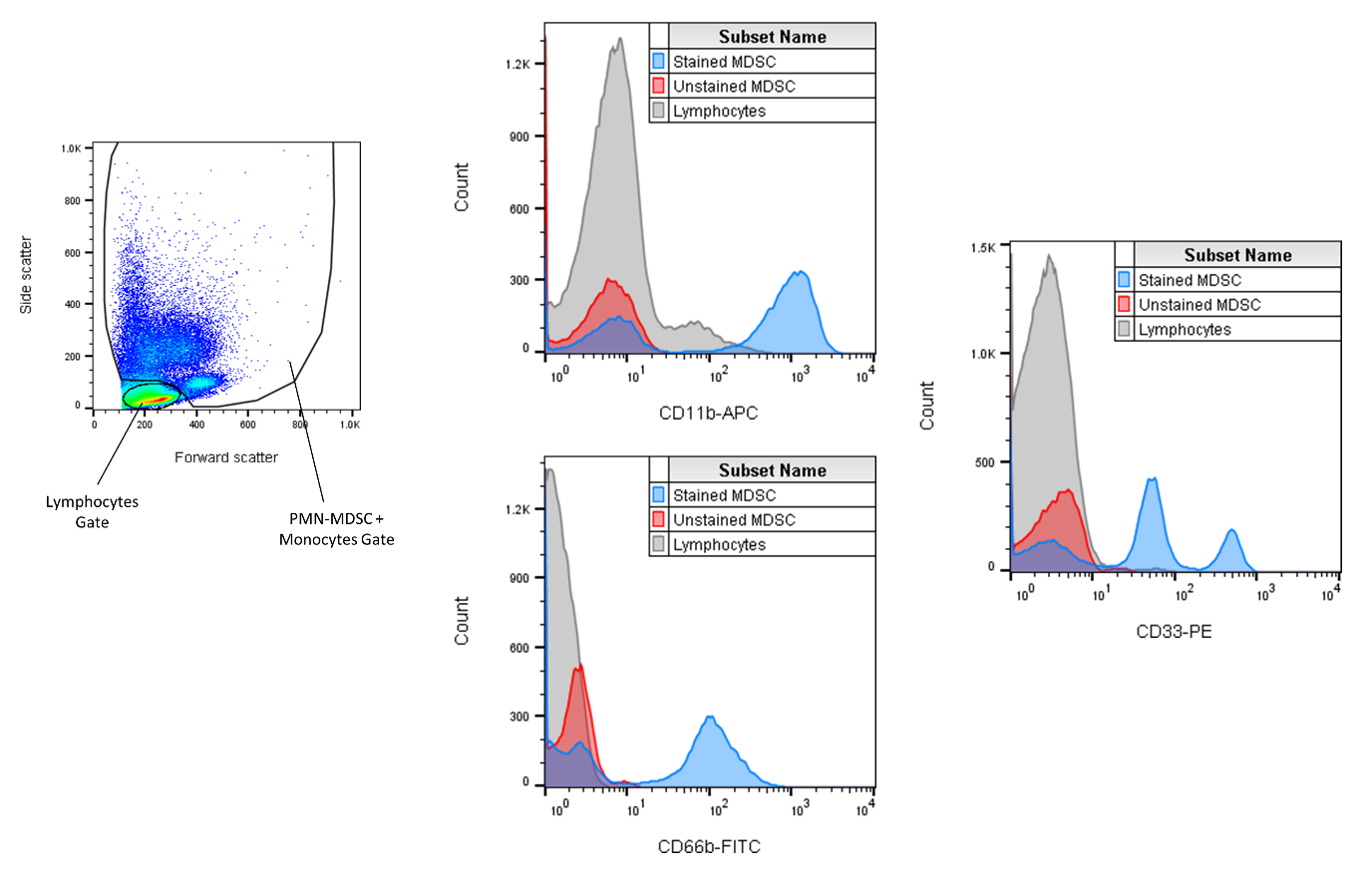


**Figure S1.** Surface markers intensity in PBMCs populations. Lymphocytes surface marker expression is represented in grey. All MDSC are located in the monocytes and PMN-like region of PBMCs. CD66b as marker for PMN-like cells is expressed in the PBMCs fraction in the SSC^hi^ reflecting the Polymorphonuclear MDSC (PMN-MDSC) population. CD33 and CD11b is expressed in PMN-MDSC and Monocytic-MDSC (M-MDSC). Unstained MDSC (in red) represents the peak for the markers in an unstained sample in the PMN-MDSC and M-MDSC region of the PBMC population.

**A**


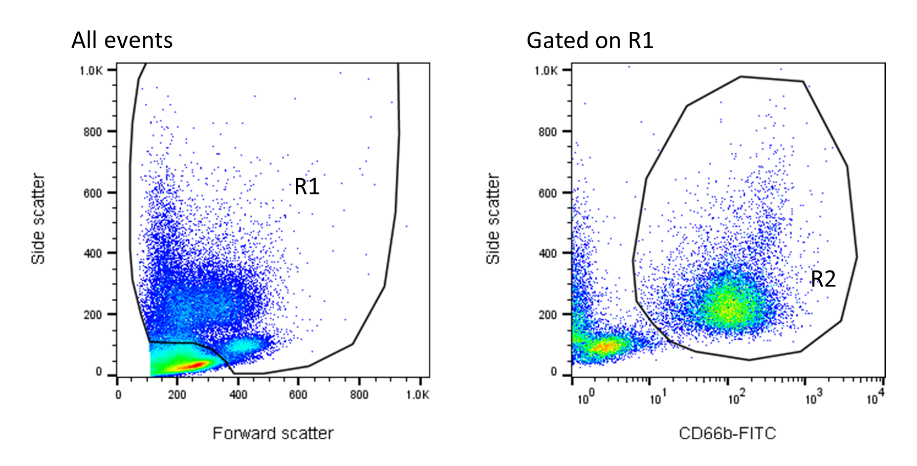


**B**


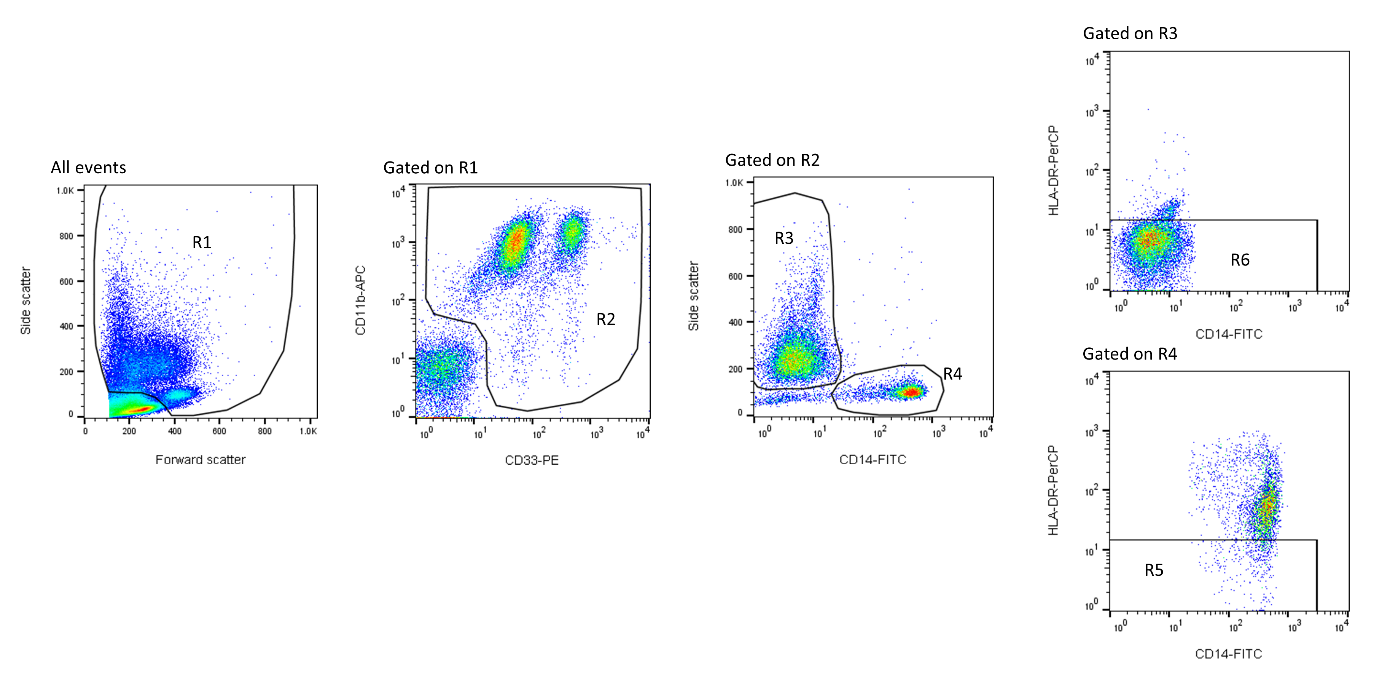


**C**


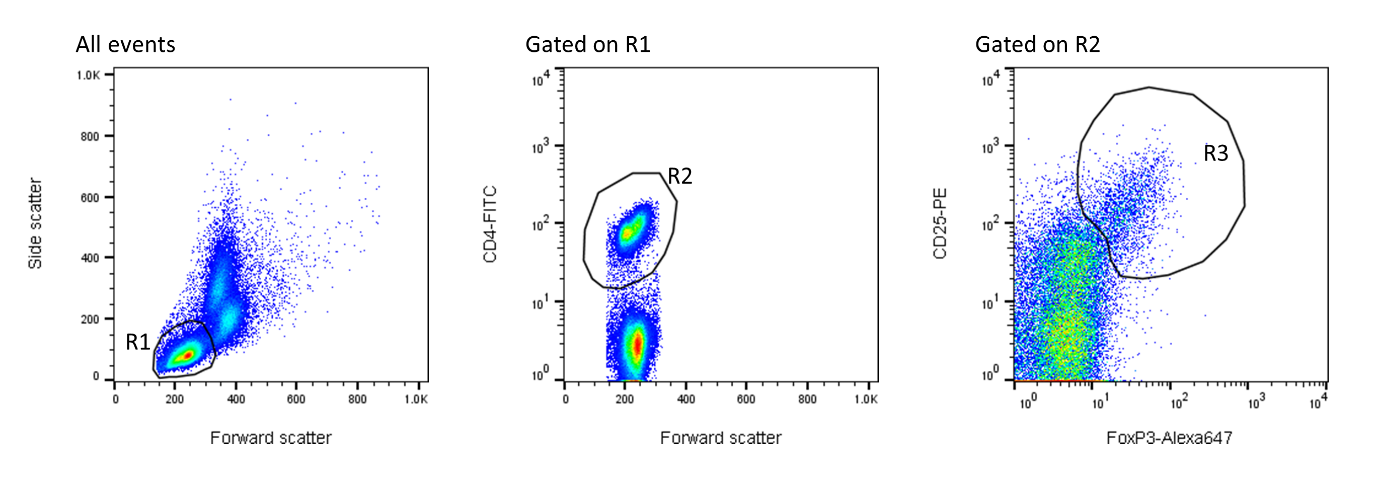


**D**


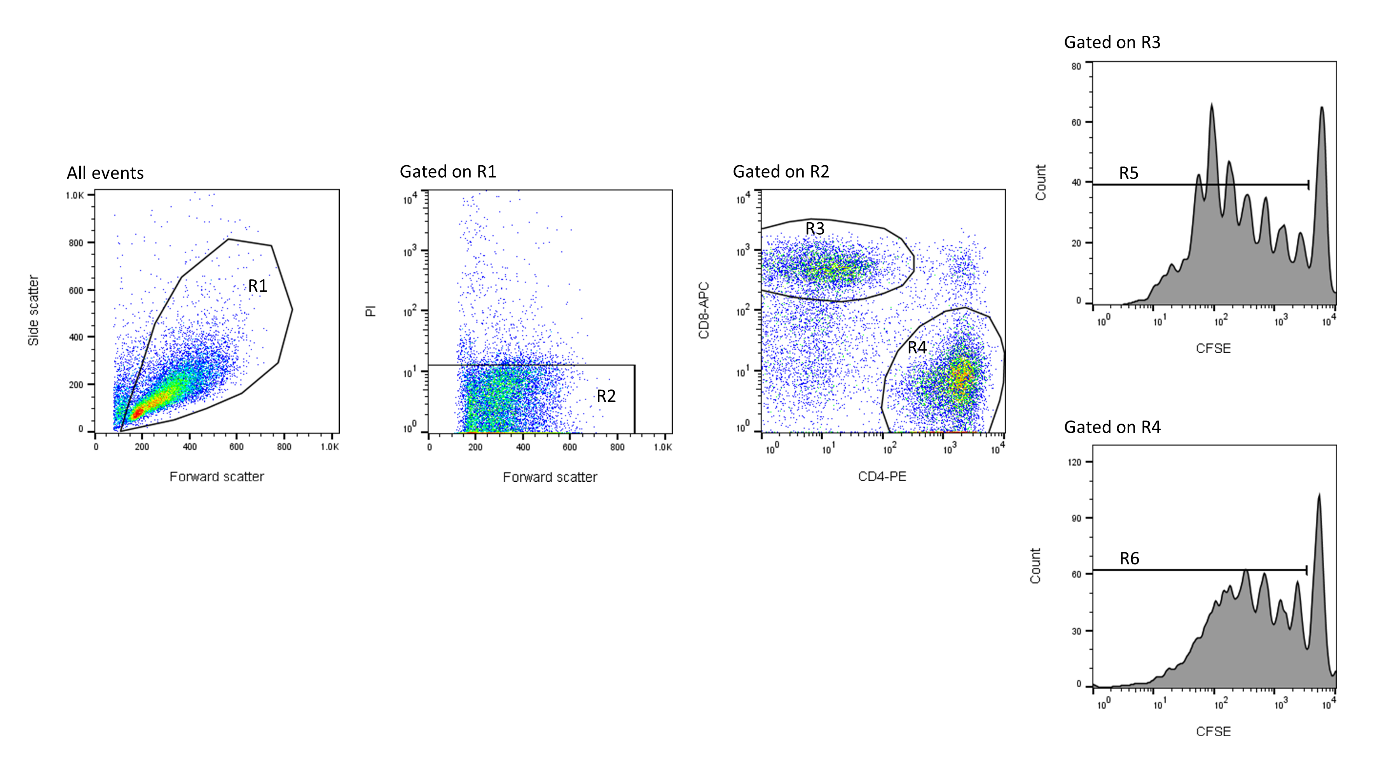


**E**

**
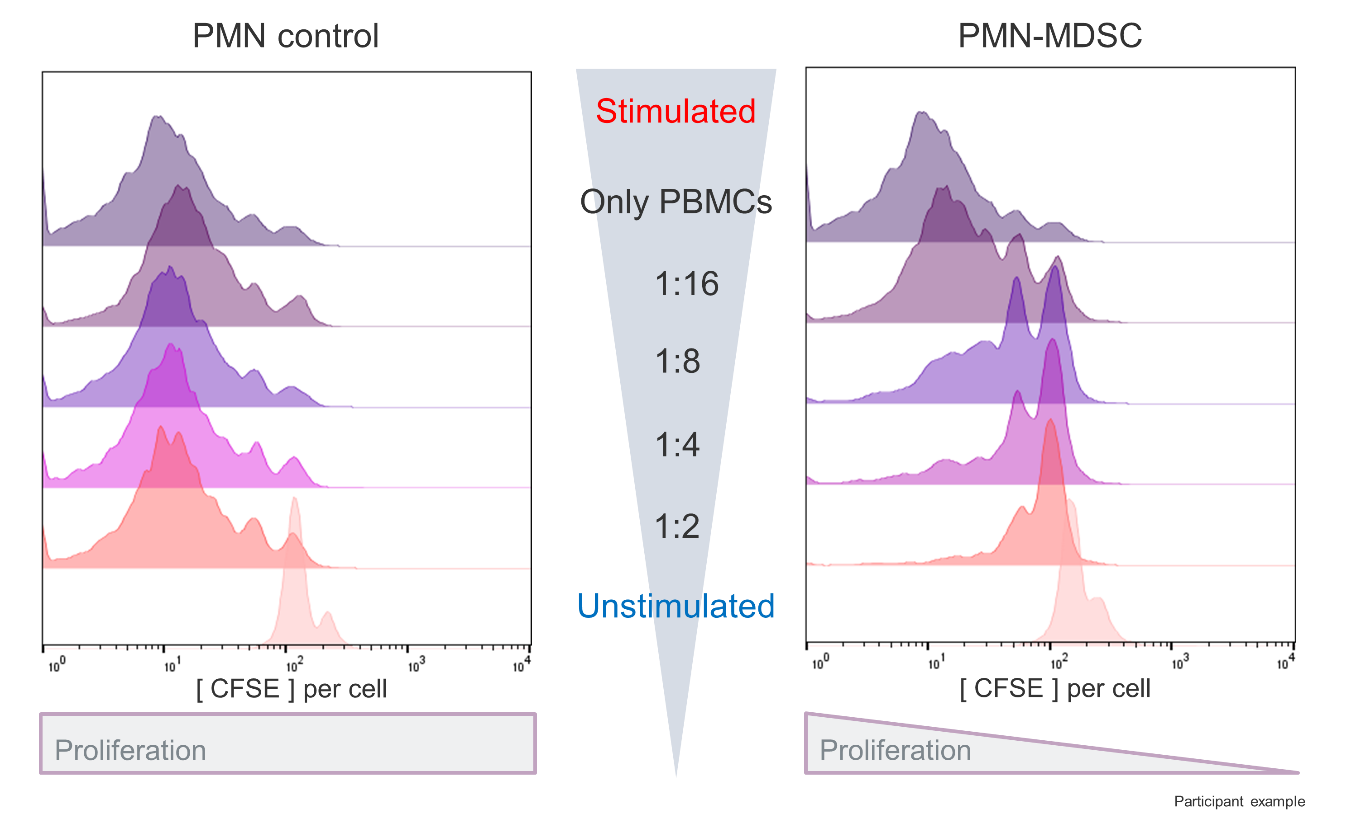
**

**Figure S2.** Gating strategy of the different studied cell populations. For all plots, one representative measurement is shown. **(A)** Polymorphonuclear MDSC (PMN-MDSC) are defined as SSC^hi^ and CD66b^+^; **(B)** Monocytic MDSC (M-MDSC) are defined as CD33^+^ CD11b^+^ CD14^+^ HLA-DR^-/lo^ in an independent sample. PMN-MDSC population can be detected as CD33^+^ CD11b^+^ CD14^-^ HLA-DR^-/lo^ in the SSC^hi^ region from the Monocytic MDSC sample; **(C)** Regulatory T cells (Treg) were discriminated from PBMCs by CD4^+^ FoxP3^+^ CD25^+/high^; **(D)** Proliferations of alive CD8^+^ (gate R3) and CD4^+^ (gate R4) cells were determined by reduction in CFSE intensity with every cell division; **(E)** Proliferations profiles of stimulated PBMC alone or in co-culture with different proportions of PMN-MDSC or PMN as negative suppression control and the expression of CFSE on the unstimulated PBMC are shown.

**A**


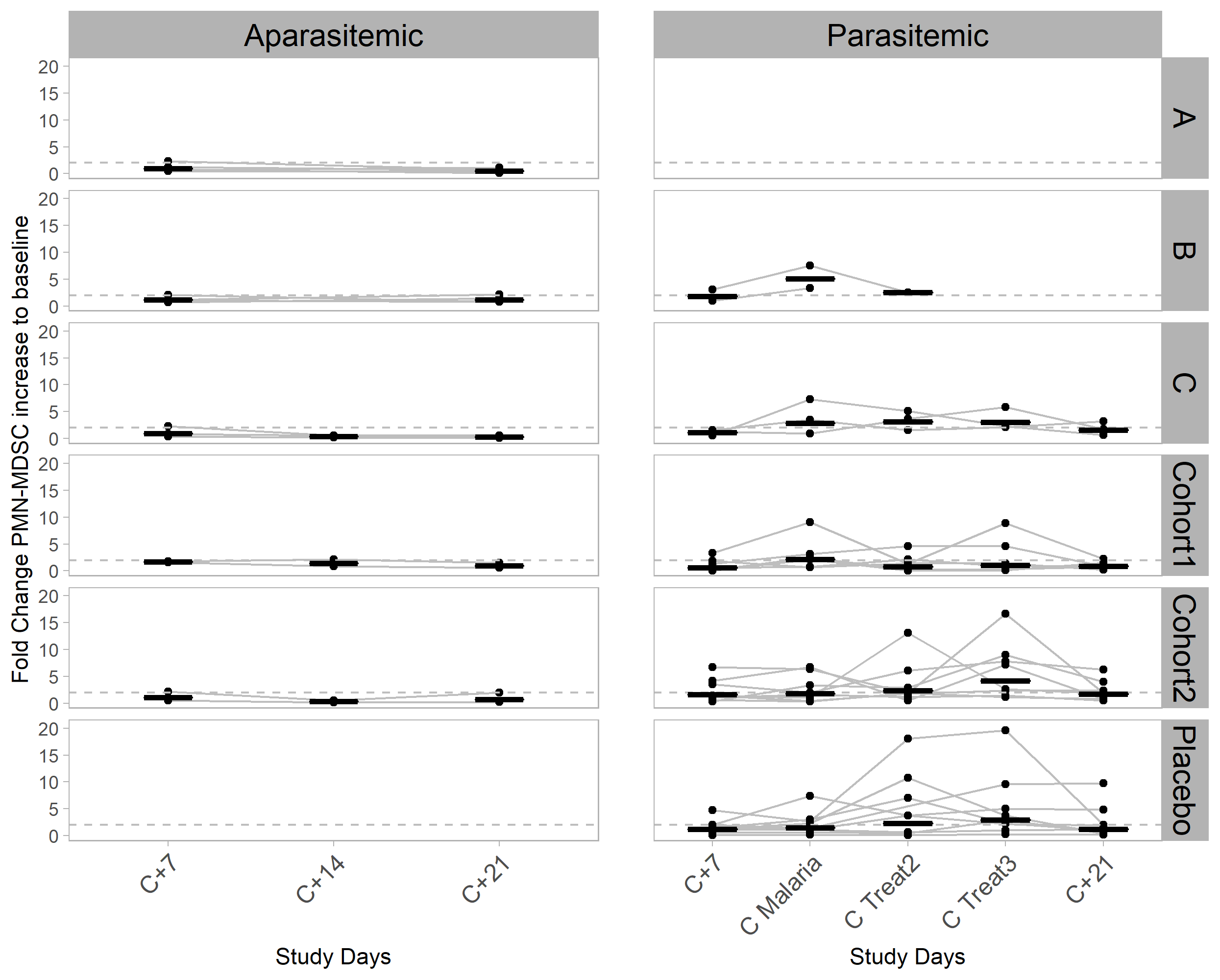


**B**


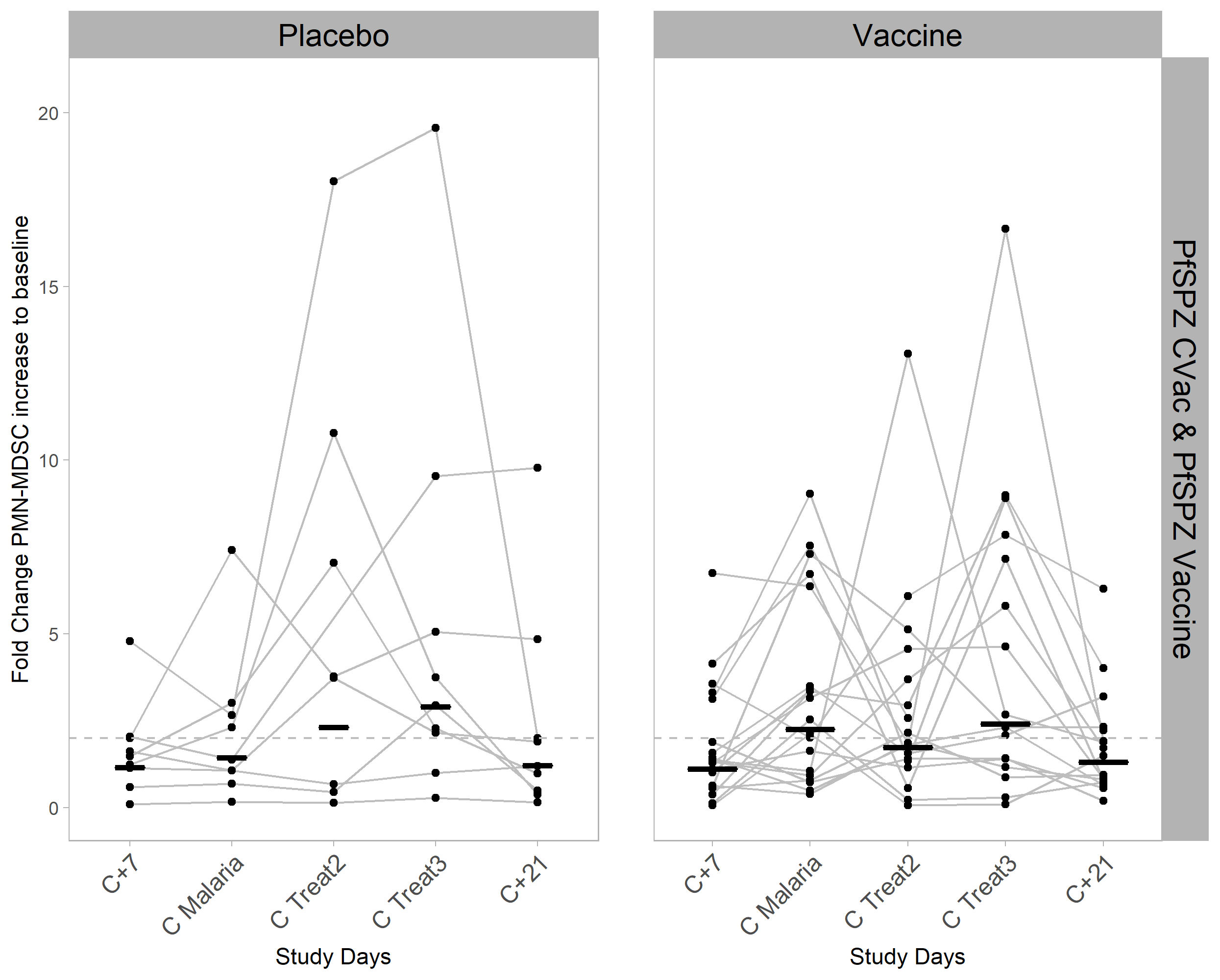


**Figure S3.** Sub analysis of PMN-MDSC kinetics over CHMI for either aparasitemic or parasitemic individuals stratified in subgroups. **(A)** No detectable difference can be seen in participants’ PMN-MDSC kinetics across the different study cohorts; **(B)** The PMN-MDSC kinetics of parasitemic participants did not differ significantly, regardless of whether they were allocated to placebo or vaccine during previous immunizations. Lines connecting dots show the variation of independent participants´ PMN-MDSC over CHMI. Short black horizontal lines represent the geometrical mean values of PMN-MDSC fold change from baseline for those time points. Dashed line represents the 2-fold change limit.
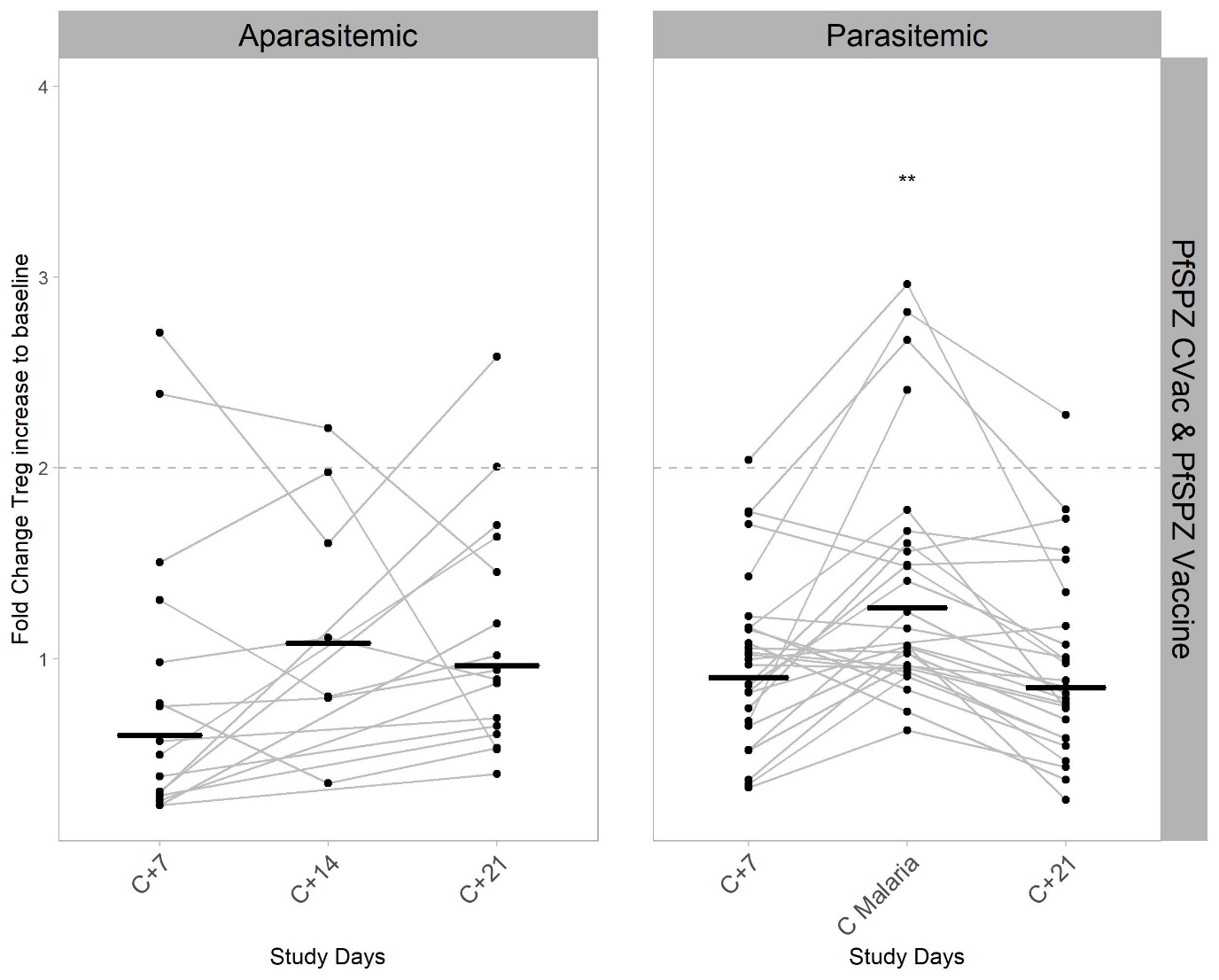


**Figure S4.** Sub analysis of T_reg_ kinetics over CHMI stratified in aparasitemic or parasitemic individuals. Significance stars (*) are given from a single analysis of parasitemic participants; ** p-value < 0.001. Lines connecting dots show the variation of independent participants´ T_reg_ over CHMI. Short black horizontal lines represent the geometrical mean values of T_reg_ fold change from baseline for those time points. Dashed line represents the 2-fold change limit.


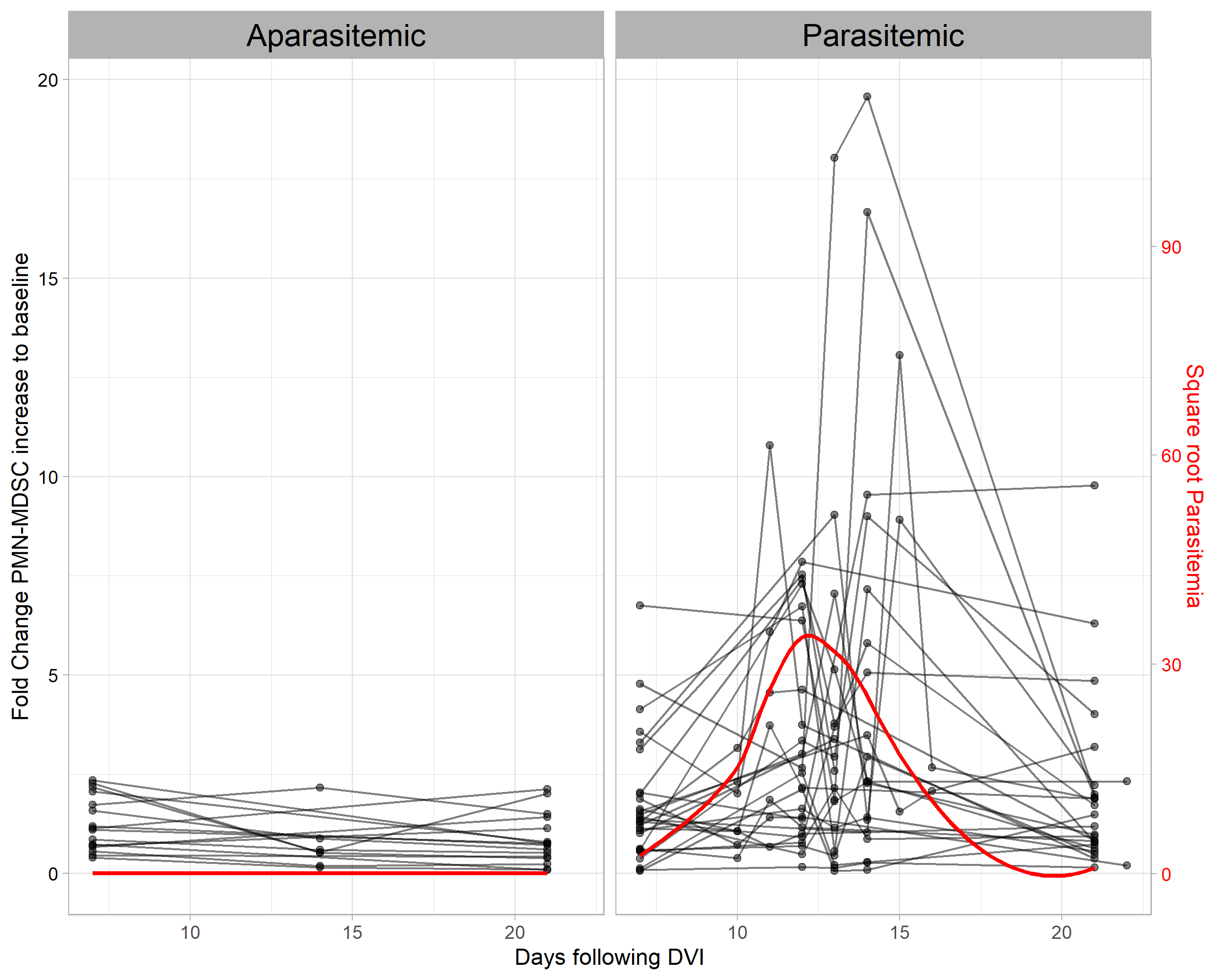


**Figure S5.** PMN-MDSC kinetics for each participant combined with the parasite blood development representation over the natural days of CHMI in aparasitemic and parasitemic individuals, independent of vaccine study allocation. The square root parasitemia over the CHMI days is plotted in form of non-linear regression for aparasitemic and parasitemic participants. Lines connecting dots show the variation of independent participants´ PMN-MDSC over CHMI.

**A**


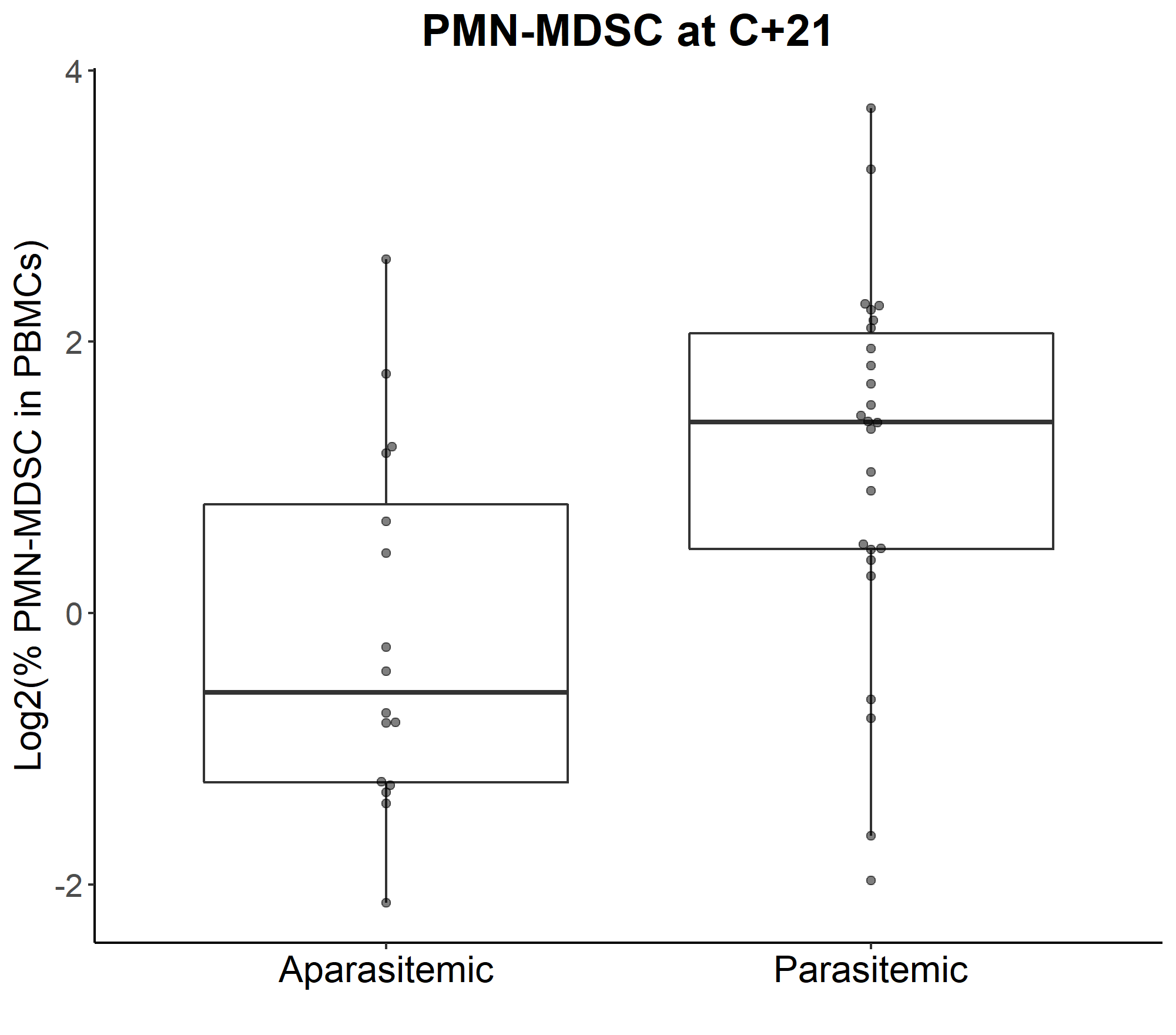


**

**B**


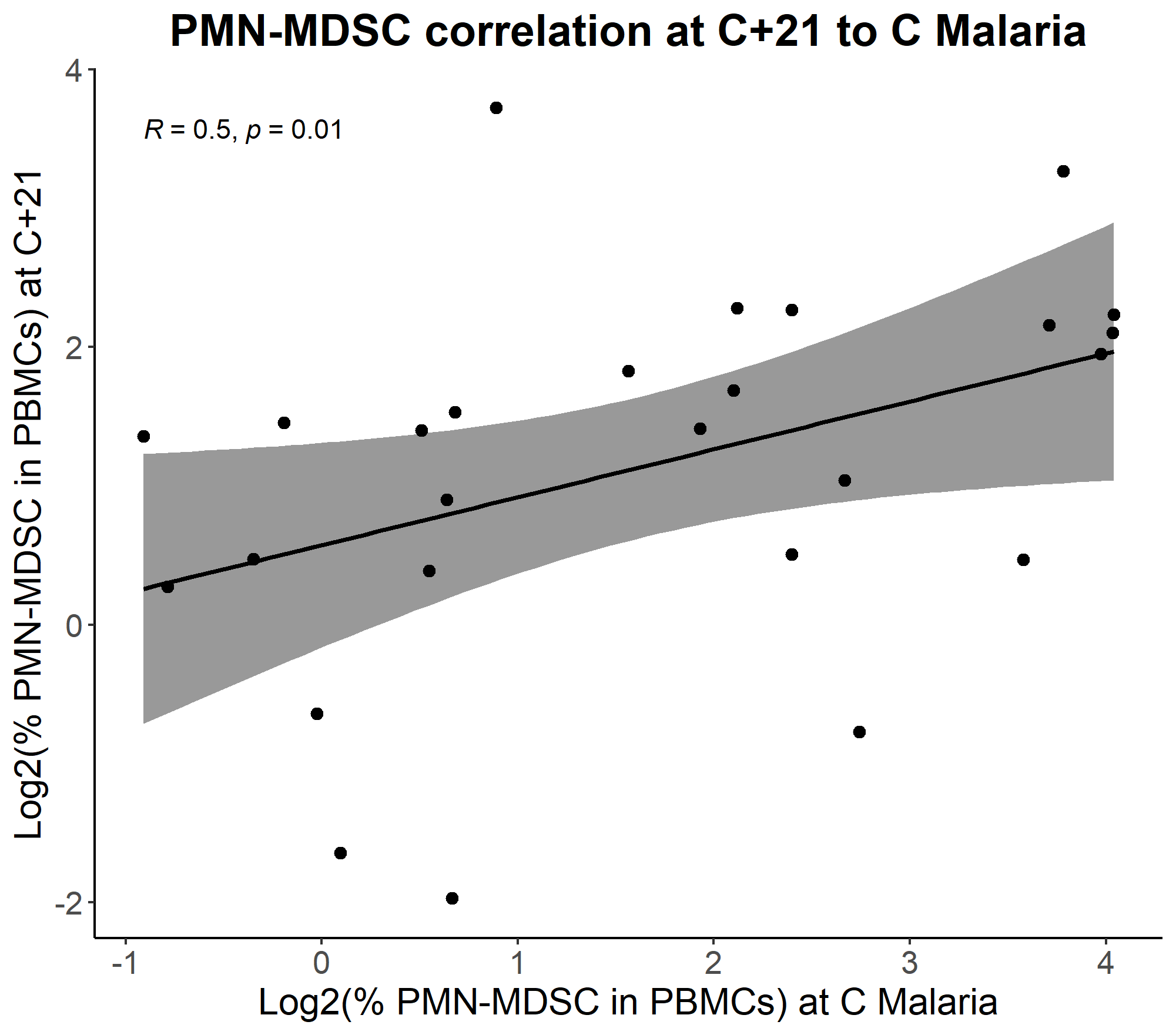


**Figure S6.** Increased PMN-MDSC on C Malaria correlate positively with the significant higher levels of PMN-MDSC in parasitemic participants at the end of the CHMI. **(A)** Boxplots showing higher % of circulating PMN-MDSC at C+21 (p-value ** < 0.005) in parasitemic participants than in the protected aparasitemic ones. **(B)** Linear model representing the positive correlation of PMN-MDSCs at C+21 with the % measured at C Malaria (Spearman's rank correlation p-value = 0.01, R = 0.5).**Table S1.** Clinical characteristics of volunteers during CHMI (study days C+7 to C+21) represented by grade 2 (moderate) and grade 3 (severe) adverse events (AE) during the time of erythrocytic stage parasitemia.

| **Parasites during CHMI** | **Preferred term** | **Severity grade** | **Volunteers with AE (n)** |
| --- | --- | --- | --- |
| Aparasitemic | Headache | Moderate | 1 |
|  | Abdominal pain | Moderate | 1 |
|  | Leukopenia | Moderate | 1 |
|  | Neutropenia | Moderate | 1 |
|  | Tachycardia | Moderate | 1 |
|  | Hyperbilirubinaemia | Moderate | 1 |
|  |  | Severe | 1 |
| Parasitemic (Unprotected) | Lymphocytopenia | Moderate | 4 |
|  |  | Severe | 2 |
|  | Headache | Moderate | 5 |
|  |  | Severe | 1 |
|  | Diastolic hypertension | Moderate | 3 |
|  |  | Severe | 3 |
|  | Thrombocytopenia | Moderate | 2 |
|  |  | Severe | 1 |
|  | Fatigue | Moderate | 2 |
|  |  | Severe | 1 |
|  | Pyrexia | Moderate | 2 |
|  | Blood pressure systolic increased | Moderate | 1 |
|  |  | Severe | 1 |
|  | Systolic hypertension | Moderate | 1 |
|  |  | Severe | 1 |
|  | Leukopenia | Severe | 1 |
|  | Neutropenia | Moderate | 1 |
|  | Angioedema | Severe | 1 |
|  | Blood creatine phosphokinase increased | Severe | 1 |
|  | Pruritus | Severe | 1 |
|  | Oropharyngeal pain | Severe | 1 |
|  | Hypertension | Severe | 1 |
|  | Urticaria | Severe | 1 |
|  | Hypersensitivity | Severe | 1 |
|  | Hypoglycaemia | Severe | 1 |
|  | Hyperglycaemia | Moderate | 1 |
|  | Nasopharyngitis | Moderate | 1 |
|  | Diarrhoea | Moderate | 1 |
|  | Abdominal discomfort | Moderate | 1 |
|  | Hypnagogic hallucination | Moderate | 1 |
|  | Vomiting | Moderate | 1 |
| Parasitemic (Placebo) | Lymphocytopenia | Moderate | 4 |
|  |  | Severe | 1 |
|  | Headache | Moderate | 3 |
|  |  | Severe | 1 |
|  | Pyrexia | Moderate | 3 |
|  |  | Severe | 1 |
|  | Diastolic hypertension | Moderate | 2 |
|  | Fatigue | Moderate | 2 |
|  | Hyperbilirubinaemia | Moderate | 2 |
|  | Leukopenia | Moderate | 2 |
|  | Abdominal pain | Severe | 1 |
|  |  | Moderate | 1 |
|  | Myalgia | Moderate | 1 |
|  | Neutropenia | Moderate | 1 |
|  | Chills | Moderate | 1 |
|  | Night sweats | Moderate | 1 |
|  | Tachycardia | Moderate | 1 |
